# Novel P2X7 Antagonist Ameliorates the Early Phase of ALS Disease and Decreases Inflammation and Autophagy in SOD1-G93A Mouse Model

**DOI:** 10.3390/ijms221910649

**Published:** 2021-09-30

**Authors:** Savina Apolloni, Paola Fabbrizio, Susanna Amadio, Giulia Napoli, Mattia Freschi, Francesca Sironi, Paolo Pevarello, Paola Tarroni, Chiara Liberati, Caterina Bendotti, Cinzia Volonté

**Affiliations:** 1 Fondazione Santa Lucia Istituto di Ricovero e Cura a Carattere Scientifico, Preclinical Neuroscience, Via del Fosso di Fiorano 65, 00143 Rome, Italy; s.apolloni.phd@gmail.com (S.A.); paola.fabbrizio@marionegri.it (P.F.); s.amadio@hsantalucia.it (S.A.); giulianapoli149@gmail.com (G.N.); 2 Laboratory of Molecular Neurobiology, Department of Neuroscience, Istituto di Ricerche Farmacologiche Mario Negri IRCCS, Via Mario Negri 2, 20156 Milan, Italy; mattia.freschi14@gmail.com (M.F.); francesca.sironi@marionegri.it (F.S.); caterina.bendotti@marionegri.it (C.B.); 3 Axxam SpA, Openzone, Via Meucci 3, Bresso, 20091 Milan, Italy; Paolo.Pevarello.PP@axxam.com (P.P.); paola.tarroni.pt@axxam.com (P.T.); chiara.liberati.cl@axxam.com (C.L.); 4 National Research Council (CNR), Institute for Systems Analysis and Computer Science “A. Ruberti”, Via Dei Taurini 19, 00185 Rome, Italy

**Keywords:** ALS, autophagy, neuroinflammation, NF-κB, NOX2, P2X7

## Abstract

Amyotrophic lateral sclerosis (ALS) is a disease with a resilient neuroinflammatory component caused by activated microglia and infiltrated immune cells. How to successfully balance neuroprotective versus neurotoxic actions through the use of anti-inflammatory agents is still under debate. There has been a boost of awareness regarding the role of extracellular ATP and purinergic receptors in modulating the physiological and pathological mechanisms in the nervous system. Particularly in ALS, it is known that the purinergic ionotropic P2X7 receptor plays a dual role in disease progression by acting at different cellular and molecular levels. In this context, we previously demonstrated that the P2X7 receptor antagonist, brilliant blue G, reduces neuroinflammation and ameliorates some of the pathological features of ALS in the SOD1-G93A mouse model. Here, we test the novel, noncommercially available, and centrally permeant Axxam proprietary P2X7 antagonist, AXX71, in SOD1-G93A mice, by assessing some behavioral and molecular parameters, among which are disease progression, survival, gliosis, and motor neuron wealth. We demonstrate that AXX71 affects the early symptomatic phase of the disease by reducing microglia-related proinflammatory markers and autophagy without affecting the anti-inflammatory markers or motor neuron survival. Our results suggest that P2X7 modulation can be further investigated as a therapeutic strategy in preclinical studies, and exploited in ALS clinical trials.

## 1. Introduction

In amyotrophic lateral sclerosis (ALS), one of the most aggressive neurodegenerative diseases of adulthood, neuroinflammation is recognized as the pathological mechanism contributing to motor neuron death [1,2,3]. In the last two decades, there has been a boost in understanding the role of extracellular ATP and purinergic signaling, i.e., the purinome dynamics [4], in the neurodegenerative/neuroinflammatory events of the CNS [5,6,7,8,9,10,11,12]. For instance, clear evidence suggests that the purinergic P2X7 receptor acts in many neurological disorders comprising ALS, to the point that it is being considered as a target for therapeutic intervention [11,13,14,15,16,17]. However, its role can be dual [13]. In particular, in P2X7^−/−^/superoxide dismutase (SOD)1-G93A mice, the most studied ALS animal model [18], the clinical onset of ALS was anticipated, and the disease progression worsened, thus suggesting that the participation of P2X7 has some beneficial function, at least in the early phases of the disease [19]. Actually, activation of P2X7 by the agonist 2′(3′)-O-(4-benzoylbenzoyl) adenosine 5′-triphosphate *in vivo*, before the onset of pathological neuromuscular features in SOD1-G93A mice, ameliorates denervation atrophy in skeletal muscles [20]. On the contrary, *in vitro* activation of P2X7 exacerbates proinflammatory responses, such as NADPH oxidase 2 (NOX2) activity, tumor necrosis factor-α (TNF-α), cyclooxigenase-2, and mitogen-activated protein kinase (MAPK) levels in ALS-microglia, and toxicity towards neuronal cells [21,22]. The deregulation of inflammatory microRNAs is also propagated by P2X7-dependent mechanisms in microglia [23,24,25]. The deleterious effects of P2X7 have lately been extended also to astrocytes [26] and to motor neurons [27]. Consistently, we and other groups have shown a certain therapeutic efficacy of a poorly centrally-penetrant P2X7 antagonist, brilliant blue G (BBG), when administered to SOD1-G93A mice [28,29,30]. In particular, we demonstrated that BBG reduces microgliosis, but not astrocytosis, modulates inflammatory transcripts, such as the nuclear factor kappa-light-chain-enhancer of activated B cells (NF-κB), NOX2, interleukin (IL)-1β, IL-10, and brain derived neurotrophic factor (BDNF). Moreover, BBG enhances motor neuron survival in the lumbar spinal cord and, finally, induces a delay of disease onset in ALS mice, however, with a modest improvement in general conditions and motor performance [29]. P2X7 antagonists that are more potent and selective than BBG, such as A804598 and JNJ-47965567, have regrettably failed under these same regards [31,32]. However, we have established that A804598 positively modulates autophagy *in vitro* and *in vivo* in SOD1-G93A models [31]. This is not surprising, because P2X7 plays a direct critical role in the regulation of the autophagic flux, and in the maturation and autophagy-based secretion of IL-1β from microglial cells [33]. Moreover, in microglia, autophagy can control innate immune functions, such as phagocytosis and inflammation [34]. Impaired autophagy is also a mechanism of injury and death in motor neurons [35], thus contributing to ALS disease progression. In particular, defects at various stages of autophagy have been consistently described as associated to the mutations of several ALS-linked genes, including *SOD1*, *SQSTM1/p62*, *TARDBP*, and *OPTN*.

In this work, we have tested new-generation P2X7 antagonists, the Axxam proprietary compound AXX71 and AXX13, with high selectivity, affinity, and blood brain barrier permeability, for their efficacy on inflammatory/autophagy mechanisms and motor skills in the SOD1-G93A mouse model of ALS.

## 2. Results

### 2.1. AXX71 Delays the Onset of Neuromuscular Impairment and Transiently Preserves Motor Abilities and Muscle Strength

AXX71 was chosen because of its *in vitro* profile was suitable for *in vivo* testing (IC_50_ values of 14 nM and 210 nM on the human and mouse P2X7, respectively, inactive on human P2Xs and P2Ys receptors and mouse P2X4, data generated by Axxam SpA, see Table 1). As it was expected by the pharmacokinetic studies, the selective and blood brain barrier permeable P2X7 antagonist, AXX71, administered daily and intraperitoneally at 30 mg/kg for two weeks reached the CNS districts, as shown by the mean concentration of the compound in the blood, brain and spinal cord of SOD1-G93A mice that resulted in 1295 ng/mL, 1102 ng/g, and 638 ng/g, respectively (Table 2).

**Figure 1:**
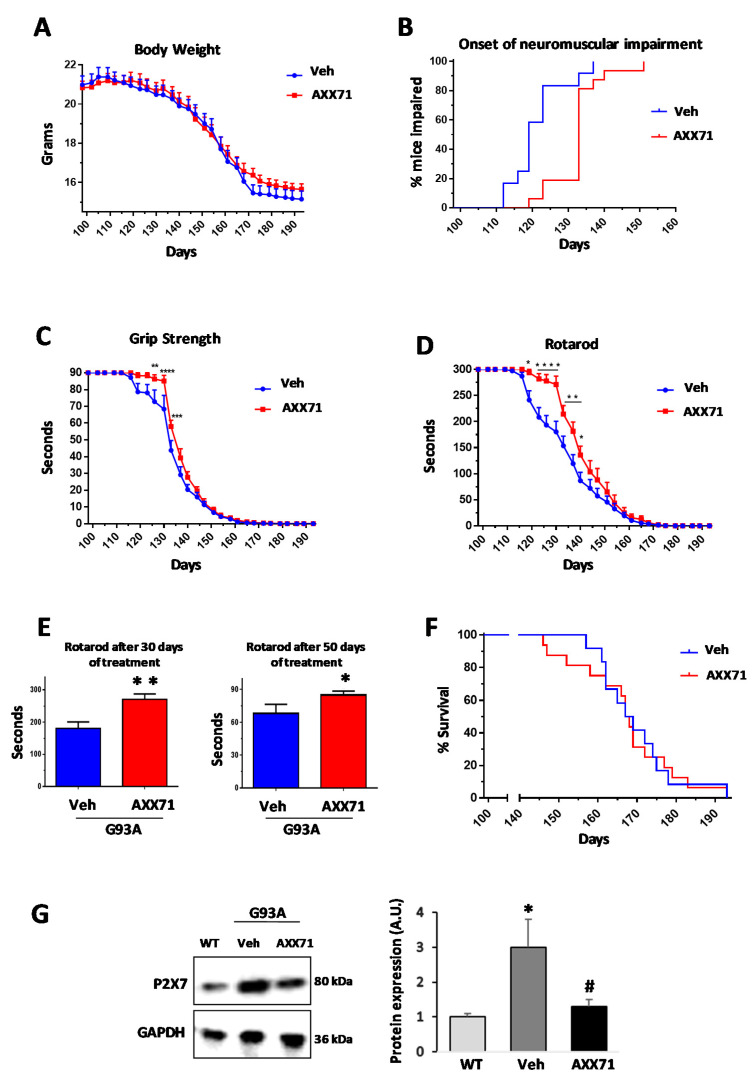
In the early stages of treatment, AXX71 maintains motor skills and muscle strength in SOD1-G93A mice. We find no significant differences in body weight (**A**) between vehicle-treated and AXX71-treated SOD1-G93A mice. AXX71 delays the onset of neuromuscular impairment (**B**) and shows transient improvement of grip strength test (**C**) and Rotarod test (**D**) in SOD1-G93A mice, in particular during the first 30 days of treatment (**E**). Kaplan–Meier survival shows no significant difference in median survival in AXX71-treated mice, with respect to vehicle-treated SOD1-G93A mice (**F**). WT group, *n* = 6 mice; vehicle- and AXX71-treated groups, *n* = 12 mice/group. (**G**) Equal amounts of total lumbar spinal cord lysates from WT mice (150 days), and vehicle-treated or AXX71-treated SOD1-G93A mice, at terminal stage (*n* = 4/group), were subjected to Western blotting with anti-P2X7 and anti-GAPDH for protein normalization. Data represent means ± SEM. Statistical significance was calculated by the log-rank test (**B**,**F**), two-way ANOVA with Sidack’s test post-analysis (**A**,**C**,**D**), or the Student’s *t*-test, (**E**,**G**), as refers to WT mice, ^#^ *p* < 0.05; or to vehicle mice, * *p* < 0.05, ** *p* < 0.01, *** *p* < 0.001, **** *p* < 0.0001.

The mean brain/blood ratio was 0.83, and the spinal cord/blood ratio was 0.49, thus indicating good CNS penetration. Moreover, AXX71 did not cause visible detrimental side effects and did not modify body weight or the motor skill parameters in wild type (WT) mice after a treatment prolonged for up to two months (not shown). We thus evaluated the efficacy of AXX71 (30 mg/kg to allow sufficient Cmax to drive activity, once a day, five days a week) on disease progression in SOD1-G93A mice, starting the treatment at post-natal day 98, i.e., 14 weeks, based on our previous encouraging results [29]. We found no significant differences between drug-treated and vehicle-treated SOD1-G93A mice in progressively losing body weight (Figure 1A). Instead, AXX71 significantly delayed the onset of neuromuscular impairment on rotarod test performance that, in 50% of mice, was retarded by about 11 days (vehicle: 121.3 ± 7.5; AXX71: 132.7 ± 7.2 mean ±SD) (Figure 1B). Moreover, AXX71 ameliorated motor performance and muscle force as assessed by the grip strength (Figure 1C) and rotarod (Figure 1D) tests. While the improvement of motor abilities and muscle strength was statistically significant compared to vehicle-treated mice during the first 30 days of treatment (Figure 1E), AXX71 failed to maintain these beneficial effects after 50 days of treatment, when the disease accelerated (Figure 1C,D), and it was totally ineffective on mice survival (Figure 1F, Table 3). Because the total level of P2X7 protein in the lumbar spinal cord of SOD1-G93A mice is known to increase with respect to the age-matched WT mice [19,36], we measured P2X7 protein expression and found that AXX71 treatment remarkably decreased it, perhaps suggesting an anti-inflammatory action exerted not only by receptor antagonism, but also by protein downregulation (Figure 1G, Table 3).

**Figure 2:**
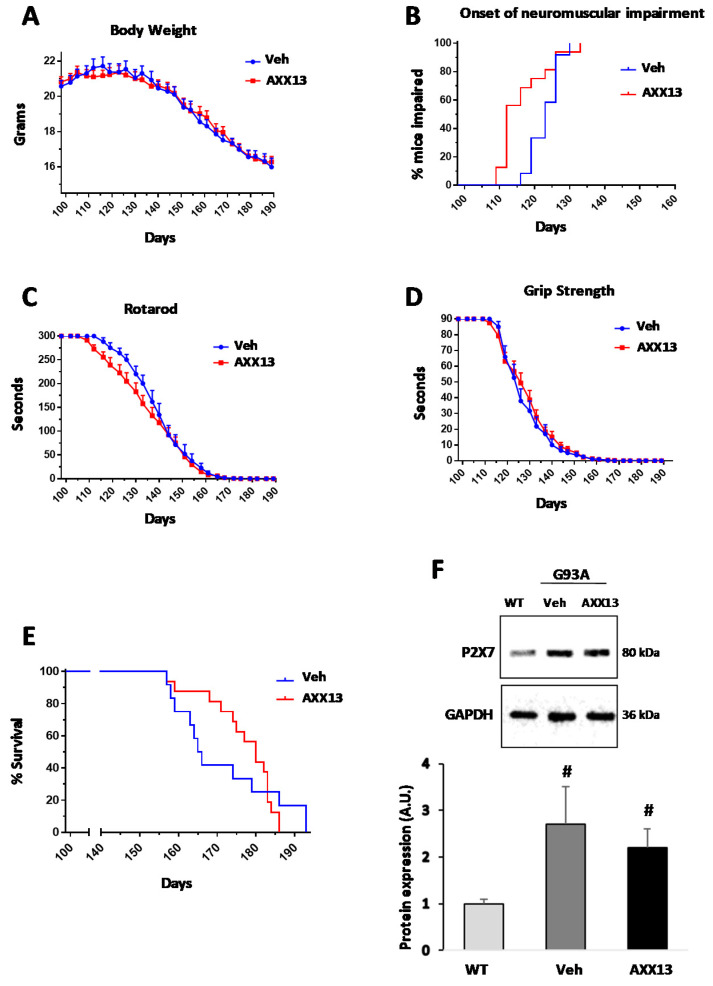
AXX13 administrated twice a day (15 mg/kg) does not improve motor abilities and muscle strength. Mice treated with the most active enantiomer of AXX71 (compound AXX13), twice a day (15 mg/kg), showed no differences in body weight (**A**), onset of neuromuscular impairment (**B**), rotarod test (**C**), grip strength (**D**), and survival (**E**), with respect to vehicle-treated SOD1-G93A mice. In (**F**), equal amounts of total lumbar spinal cord lysates from WT mice (150 days) and vehicle-treated, or AXX13-treated, SOD1-G93A mice, in the terminal stage (*n* = 4/group), were subjected to Western blotting with anti-P2X7 and anti-GAPDH for protein normalization. Data represent means ± SEM. Statistical significance was calculated by the log-rank test (**B**,**E**) two-way ANOVA with Sidack’s post-analysis (**A**,**C**,**D**) or the Student’s *t*-test, (**F**) as refers to WT mice, ^#^ *p* < 0.05.

Furthermore, this mechanism seems quite significant because a different protocol, administering the most active enantiomer of AXX71 (compound AXX13, IC_50_ values of 6 nM and 109 nM on human and mouse P2X7, respectively, and inactive on human P2Xs and P2Ys receptors and mouse P2X4, data generated by Axxam SpA, see Table 1) at the lower dose of 15 mg/kg intraperitoneally, but twice a day, with the aim of prolonging target coverage (Figure 2), did not cause P2X7 protein downregulation (Figure 2F), but it was unable to sustain the transitory improvement in motor abilities and muscle strength (Figure 2A–D). In addition, AXX13 elicited a trend in improving survival (Figure 2E) that did not reach statistical significance, but that would certainly need further investigation. Similarly, the P2X7 antagonist, A804598, failed to affect P2X7 expression levels in the spinal cord or to ameliorate disease progression (Table 3).

### 2.2. AXX71 Does Not Affect Motor Neuron Survival and Gliosis, but Decreases Proinflammatory Markers and NF-κB in SOD1-G93A Spinal Cord

**Figure 3:**
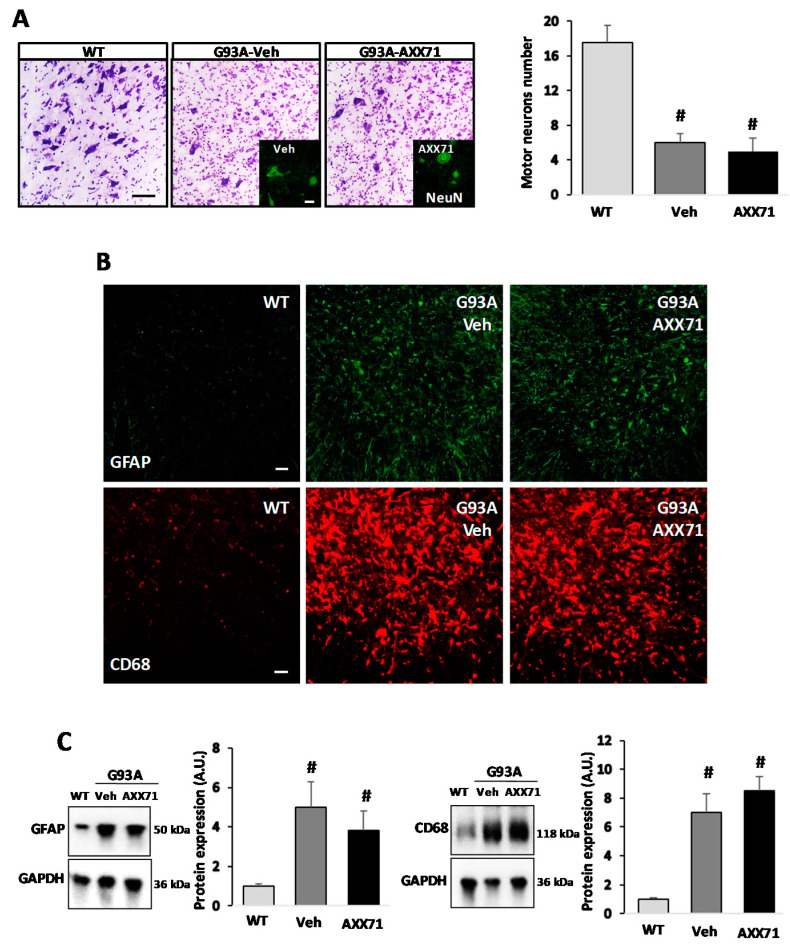
AXX71 treatment does not decrease motor neuron loss and gliosis in lumbar spinal cord of SOD1-G93A mice. (**A**) Spinal cord sections (L3–L5) from WT mice (150 days), and terminal stage SOD1-G93A mice after vehicle or AXX71 30 mg/kg treatment, were stained with cresyl violet. Scale bar: 50 μm. The inserted image is representative of anti-NeuN staining (green). Scale bar: 10 μm. Assessment of motor neuron number was performed by direct counting (*n* = 4/group). (**B**) Representative confocal images of lumbar spinal cord sections of WT mice, vehicle-treated, and AXX71-treated SOD1-G93A mice immunolabelled with anti-GFAP (green), and anti-CD68 (red). Scale bar: 50 μm. (**C**) Equal amounts of total lumbar spinal cord lysates from WT mice (150 days), and vehicle-treated or AXX71-treated SOD1-G93A mice, at terminal stage (*n* = 4/group) were subjected to Western blotting with anti-GFAP, anti-CD68, and anti-GAPDH for protein normalization. Data represent means ± SEM. Statistical significance was calculated by one-way ANOVA with Sidack’s post-analysis or Student’s *t*-test, as refers to WT mice, ^#^ *p* < 0.05.

We next tested motor neuron survival and neuroinflammation in the ventral horns of the lumbar spinal cord from AXX71-treated, compared to vehicle-treated, SOD1-G93A mice at the end stage of the disease. AXX71 neither prevented the loss of Nissl substance in ALS motor neurons, nor rescued the average number of healthy motor neurons (Figure 3A, vehicle: 6.1 ± 0.8 vs. AXX71: 5.3 ± 1.2 motor neurons/hemisection/mouse). However, a reduction in the number of motor neurons was evident and significant when comparing SOD1-G93A mice and WT mice (*p* < 0.05). Neither astrogliosis nor microgliosis were restrained by AXX71, as evaluated by GFAP and CD68 confocal immunofluorescent staining and Western blotting (Figure 3B,C). In a similar way, neither A804598 nor AXX13 treatments affected motor neuron loss or astrogliosis and microgliosis in the SOD1-G93A spinal cord (Table 3).

**Figure 4:**
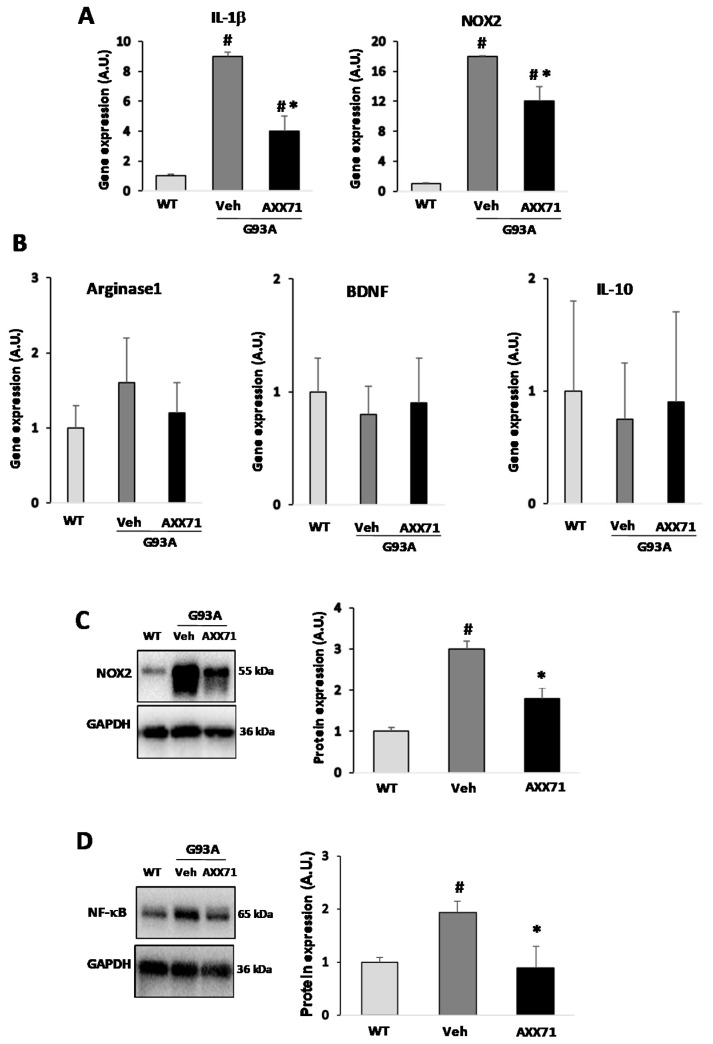
AXX71 treatment reduces inflammatory markers in the spinal cord of SOD1-G93A mice. RNA was extracted from lumbar spinal cords of WT mice (150 days), and vehicle-treated or AXX71-treated SOD1-G93A mice at terminal stage. The expression profiles of IL-1β and NOX2 (**A**) and of Arginase-1, BDNF and IL-10 (**B**) were examined by qRT-PCR. Equal amounts of total lumbar spinal cord lysates from WT mice (150 days), and vehicle-treated or AXX71-treated SOD1-G93A mice, at terminal stage (*n* = 4/group), were subjected to Western blotting with anti-NOX2 (**C**) or anti-NF-κB (**D**) and anti-GAPDH for protein normalization. Data represent means ± SEM. Statistical significance was calculated by one-way ANOVA with Sidack’s post-analysis or Student’s *t*-test, as refers to WT mice, ^#^ *p* < 0.05; or to vehicle mice * *p* < 0.05.

Interestingly, AXX71 treatment downregulated the microglia proinflammatory M1-like markers IL-1β and NOX2 mRNA expression in SOD1-G93A with respect to vehicle mice (Figure 4A), although the antagonist was ineffective in modulating the anti-inflammatory M2-like markers Arginase-1, BDNF and IL-10 mRNA content (Figure 4B). Future experiments might consider investigating these same markers at the symptomatic phase of the disease. The downregulation of the key players of inflammation, NOX2 and NF-κB, in AXX71-treated, compared to vehicle-treated, SOD1-G93A mice was confirmed by a Western blot analysis of the total protein extracts from the lumbar spinal cord (Figure 4C,D). It would be of interest to also investigate the effects of AXX71 treatment on the activated forms of the key transcription factors involved in the disease, beginning with pNF-κB. Compound AXX13 was totally ineffective in modulating inflammatory markers, while A804598 downregulated only IL-1β mRNA expression (Table 3).

### 2.3. AXX71 Modulates Autophagic Markers in SOD1-G93A Mice

**Figure 5:**
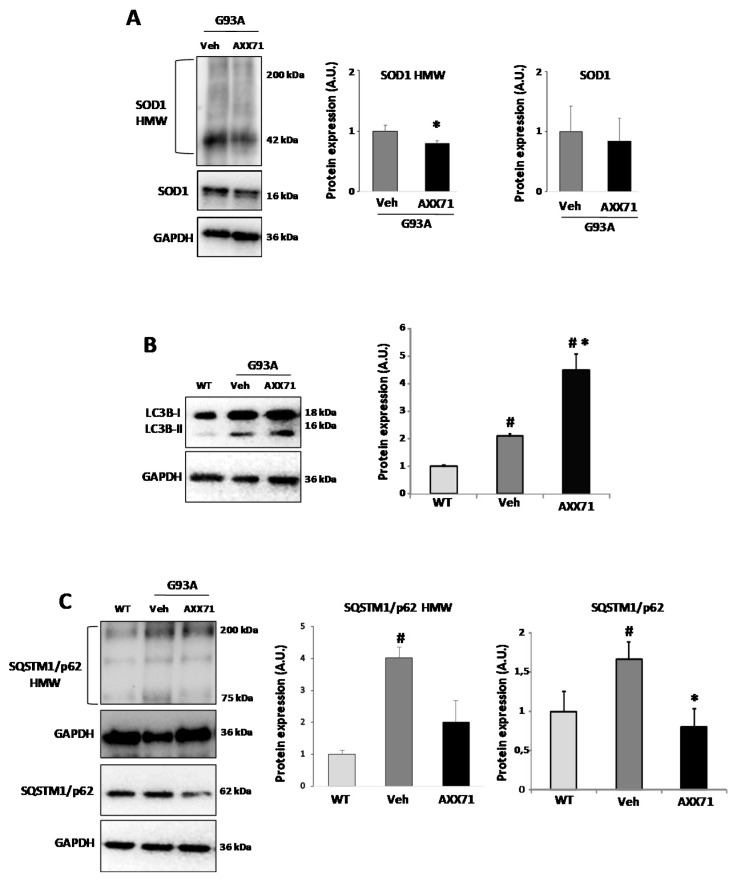
AXX71 treatment modulates autophagic markers. Equal amounts of total lumbar spinal cord lysates from WT mice (150 days), and vehicle-treated or AXX71-treated SOD1-G93A mice, at terminal stage (*n* = 4/group), were subjected to Western blotting with anti-SOD1 ((**A**) under nonreducing conditions), anti-LC3BI-II (**B**) anti-SQSTM1/p62 (**C**) (under reducing or nonreducing conditions for the detection of monomers and oligomers, respectively) and anti-GAPDH for protein normalization. SOD1, LC3-II, and SQSTM1/p62 were quantified and normalized to GAPDH protein levels. H*M*_W_ = high molecular weight. Data represent means ± SEM. Statistical significance was calculated by one-way ANOVA with Sidack’s post-analysis or Student’s *t*-test, as refers to WT mice, ^#^ *p* < 0.05; or to vehicle mice * *p* < 0.05.

We next studied the effect of the AXX71-dependent antagonism of P2X7 on SOD1 expression and aggregation, and autophagy, by measuring the levels of SOD1, LC3B-II, and SQSTM1/p62 proteins. AXX71 treatment partially decreased the levels of high molecular weight SOD1 aggregates, without reducing the amount of monomeric SOD1 protein in the lumbar spinal cord of SOD1-G93A mice with respect to the vehicle group (Figure 5A). Moreover, in accordance with previous studies [31,37], the levels of LC3B-II and SQSTM1/p62 proteins were both increased in end-stage vehicle-treated SOD1-G93A mice with respect to the WT mice, indicating impairment of the autophagy machinery. Remarkably, LC3B-II was significantly further increased (Figure 5B) while both the monomeric, as well as the oligomeric forms (although this latter not reaching the statistical significance, *p* = 0.06) of SQSTM1/p62 were decreased in ALS mice by AXX71-treatment (Figure 5C), thus suggesting that the compound was able to partially restore autophagy defects, probably by degrading SQSTM1/p62 protein [38].

## 3. Discussion

Proceeding from the initial recognition of the contribution of extracellular ATP to the neurodegeneration of the nervous system [10], to the encouraging results on the role of P2X7 in ALS [11,12], the present study aimed to provide a further translational opportunity in ALS by exploiting the therapeutic efficacy of new noncommercially available P2X7 antagonist AXX71 and AXX13 compounds. Although mutations in the *SOD1* gene have been found in approximately 15% of familial ALS patients, and about 1% of sporadic ALS patients, the model used in this study-the transgenic SOD1-G93A mouse-represents the one most extensively characterized, because it recapitulates many features of the sporadic ALS phenotype. Indeed, except for the age of onset, the clinical features of sporadic and familial ALS are indistinguishable during disease progression, as some biomarkers are [39]. Thus, in our study, the rationale for starting the treatments at 14 weeks of age in SOD1-G93A mice resides in the belief that this time window of pharmacological intervention could strongly improve the translational impact of the results, providing the basis for possible future trials in ALS patients, and particularly those affected by a sporadic form of the disease, whose treatment cannot be initiated before the onset of symptoms.

We demonstrated that the AXX71 (but not AXX13) compound is efficacious in temporarily preserving motor abilities and muscle strength in SOD1-G93A mice, and in counteracting some ALS features. However, when the disease accelerates, the initially positive outcomes of AXX71 gradually diminish, without finally producing beneficial effects on survival. With no doubt, these results further support the involvement of P2X7 in ALS, as confirmed by other scientists [28,29,30,40] and, at the same time, reinforce the dual role that P2X7 plays in the disease [13]. Moreover, in the present work, we have shown that AXX71 downregulates P2X7 protein, eliciting temporarily beneficial effects when provided once a day, while AXX13 administered twice a day does not modulate the receptor and is not protective by any means. Although we think that P2X7 levels are not directly linked to the severity of ALS symptoms, this is surely a matter to be debated and further investigated. Indeed, neither the nonspecific antagonist BBG, ameliorating disease progression to various extents [28,29], nor the most specific JNJ47965567 under protective [41] or nonprotective conditions [32], can modulate P2X7 mRNA/protein content in the lumbar spinal cord of SOD1-G93A mice. On the other hand, the anti-inflammatory clemastine can confer neuroprotection in ALS mice under conditions of either P2X7 protein upregulation (if administered for a limited time during the asymptomatic phase) [42], or downregulation (if administered at the early symptomatic until end stage) [43]. In other words, we still don’t know how a modulated expression of P2X7 is directly linked to ALS pathology and, moreover, why AXX13 therapeutically failed, despite a potency profile higher than AXX71, and a more frequent administration protocol. An educated guess would suggest that inhibiting P2X7 is a good practice in ALS, but within a restricted time window and by limited dosage during disease progression. Indeed, by sustaining the “too much/too bad inhibition” hypothesis, total ablation of the receptor is deleterious [19], as the more frequent administration of the most potent AXX13 is pejorative with respect to AXX71.

Next, we proved that AXX71 treatment significantly downregulates proinflammatory IL-1β, NOX2, and NF-κB expression in the SOD1-G93A spinal cord at the end stage of disease and, concomitantly, modulates autophagic LC3B-II and SQSTM1/p62 proteins (Table 3). This encouraging evidence establishes that a treatment with AXX71 that is prolonged up to later time points, is effective on the signal transduction mechanisms sustaining neuroinflammation and autophagy, while is apparently ineffective on motor abilities at later stages, motor neuron survival, and mice lifespan. P2X7 has been previously reported to negatively regulate autophagy by impairing lysosomal function in mouse microglia and human epithelial cells [44]. Conversely, P2X7 increases autophagy in monocytes and macrophages during mycobacterial infections [45], acting also as a positive autophagy regulator in dystrophic muscles [46]. We have previously demonstrated that, in SOD1-G93A primary microglia, activation of P2X7 does indeed deregulate the LC3-II and p62 autophagic markers, and these effects are prevented by the selective P2X7 antagonist, A-804598 [31]. Most importantly, A804598 also decreases the autophagic substrate, p62, accumulating *in vivo* as a consequence of autophagy impairment, as we have now confirmed here with AXX71. It will be of interest to further establish if, and how, AXX71 affects the autophagic flux and protein aggregation in the different cell phenotypes that are involved in ALS. 

Overall, these results confirm that the context- and time-specific involvement of P2X7 in ALS extends to the neuroinflammatory and autophagic mechanisms, thus suggesting that the lead antagonist, AXX71, deserves further investigative efforts. Because of its favorable action in ameliorating some ALS features, AXX71 can be added to the list of those P2X7 antagonists [47] that, with time, will assist in dissecting the pathogenic marks of ALS. It also renews our hope for an efficacious P2X7-dependent treatment by showing that, in the search for a combined therapy for ALS, neuroinflammation and autophagy are already P2X7-defeated pathways.

As expected, these findings also open a new scenario for questions. For instance, it will be fundamental to understand: (i) the cause-effect relationships between neuroinflammation/autophagy and those additional, still unknown, pathways that are perhaps under the control of P2X7 in ALS; (ii) the specific contribution to ALS of each P2X7-bearing cell phenotype and, particularly, the efficacy of a central versus peripheral cell-specific modulation of P2X7; (iii) the most appropriate timing and dosing for improving the therapeutic effectiveness in ALS through early versus late P2X7 modulation; and (iv), the role possibly played by P2X7 in additional ALS models.

As previously reported by various groups, some selective CNS-penetrant antagonists seem to be less efficacious than the poorly selective and blood brain barrier permeable P2X7 antagonist, BBG. For instance, the potent JNJ-47965567 [48] totally failed in altering the survival, disease progression, and the molecular and cellular parameters of ALS in SOD1-G93A mice when compound administration was performed three times per week from the early symptomatic phase [32]. Similar results were observed with the A804598 antagonist [31]. However, when JNJ-47965567 was injected in ALS mice four times per week, starting at post-natal day 60, the antagonist was able to delay disease onset, reduce body weight loss, and improve the motor parameters, although without affecting the survival or the motor neuron number [41]. Among the possible reasons is that P2X7 could be centrally but favorably “less inhibited” by the poor CNS-permeable BBG during the pre/early symptomatic phase of ALS, when the most potent and CNS-penetrant antagonists are generally provided, and when the onset of ALS is even anticipated by the genetic deletion of the receptor. By confirming the dual, early-beneficial and late-detrimental role possibly played by P2X7 in ALS, we have recently demonstrated that the direct activation of the receptor by the agonist 2′(3′)-O-(4-benzoylbenzoyl) adenosine 5′-triphosphate during the pre-symptomatic phase is ameliorative by inducing the improvement of the innervation and metabolism of the myofibers, the proliferation/differentiation of the satellite cells, and the prevention of the denervation atrophy of skeletal muscles in SOD1-G93A mice [20]. In addition, BBG could also block the P2X7-dependent detrimental mechanisms on the peripheral immune cells, where the more centrally penetrant P2X7 antagonists are less effective. All this considered, there are good reasons for prospecting the clear benefits of a P2X7-oriented approach for clinical translation, but there is also further research to be promoted, and many challenges to be solved. 

In conclusion, these observations substantiate the hypothesis that P2X7 acts not only in the central, but also in the peripheral, pathways of ALS. Hopefully, this proposition will receive further reinforcement in the near future after finalization of the design (centrally/peripherally oriented), and time-dependent (early/late symptomatic), administration protocols with the most promising pharmacokinetic and pharmacodynamic P2X7 antagonists, comprising, of course, AXX71. Considering that only two drugs are commercially available at present against ALS, the glutamate antagonist Riluzole, and the antioxidant Edaravone, with scarce effects in delaying disease progression [49], and also that ALS is multigenic and multifactorial [50], we believe that positive outcomes in mouse models and clinical trials might be obtained by adopting multitarget approaches. In this context, our present results support the idea that ALS proceeds through several mechanisms in which P2X7 might also play a key role.

## 4. Materials and Methods

### 4.1. Animal Model

Procedures involving animals and their care were conducted according to the Mario Negri Institute and the Fondazione Santa Lucia institutional guidelines, in compliance with national regulations (D.lgs 26/2014) and policies providing authorization for persons conducting animal experiments (Quality Management System certificate-UNI EN ISO9001:2008–reg.N6121), with the NIH Guide for Care and Use of Laboratory Animals (2011 edition) and, finally, with EU directives and guidelines (EEC Council Directive 2010/63/UE). All experiments and protocols were examined by Institutional Ethical Committees and received authorization from the Italian Ministry of Health (Authorization n.783/2016-PR for Mario Negri Institute, and n.319/2015PR for Fondazione Santa Lucia). The animals were housed under specific pathogen-free standard conditions (22 °C ± 1 °C, 55% ± 10% relative humidity, and 12 h light/dark schedule), 3–4 per cage, with free access to food (standard pellet, Altromin, MT, Rieper) and water. Animals with motor impairment received food on the cage bottom, and water by bottles with long drinking spouts.

Given the sex-dependent effects exerted by drugs in SOD1 models [51] and, in particular, the interesting effects exerted by BBG on female SOD1-G93A mice [28,52], and the various controversial male vs. female differences encountered with P2X7 antagonists or genetic ablation [52], we decided to use only females in order to avoid gender diversity and compare our previous results with BBG. Female transgenic B6.Cg-Tg(SOD1*G93A)1Gur/J (SOD1-G93A), and non-transgenic female littermates C57BL/6J mice (wild type, WT), were originally obtained from Jackson Laboratories (Bar Harbor, ME, USA) and maintained on a C57BL6/J background at the AriSLA animal facility of the Mario Negri Institute, Milan, and Fondazione Santa Lucia, Rome. Hemizygous mice exhibit a sick phenotype similar to ALS in humans, becoming paralyzed in one or more limbs, with the paralysis due to loss of motor neurons in the spinal cord. Transgenic mice have abbreviated life spans, with the average death at 169 ± 15 days. The disease end stage is defined as the day when the mouse is unable to turn itself within 10 s, after being placed on either side. This time point is considered as the index of survival length. Disease duration is calculated as the difference in days between the onset and the end stage.

### 4.2. Drug Treatments

The proprietary P2X7 antagonist, AXX71 (PCT Int Appl. WO2015118019A1, Axxam SpA), was kindly provided by Axxam SpA. After confirming the stability of the compound in 30% Captisol^®^ solution for up to 14 days, all mice were treated daily (5 times a week), and intraperitoneally, with AXX71 at 30 mg/kg in 30% Captisol^®^ solution (AXX71-SOD1-G93A *n* = 16, AXX71-WT *n* = 6), or 30% Captisol^®^ solution alone (vehicle-SOD1-G93A *n* = 12), starting from 14 weeks of age, i.e., when the mice show the first signs of symptoms (hind limb tremors), until death. In a second set of experiments, SOD1-G93A mice (*n* = 12) were treated twice a day intraperitoneally with the active enantiomer of AXX71, compound AXX13, at 15 mg/kg in 30% Captisol^®^ solution. This experimental protocol was justified by the need to further increase receptor occupancy while remaining within the 30 mg/kg daily. It is important to note that the vehicle used for these treatments did not alter the behavioral features (Supplementary Table S1), nor the biochemical characteristics, of the SOD1-G93A mouse colony (Supplementary Table S2).

### 4.3. Analysis of Motor Dysfunction and Survival

The mice groups were randomized to equilibrate the variability based on the initial body weight and motor performance before the treatments (littermates were equally distributed in different groups). All animals were tested twice weekly, starting from the first day of treatment. An operator who was blind to the mice treatments evaluated the deficits in grip strength and rotarod performance. The body weight was also evaluated for each animal. To test grip strength, mice were placed on a horizontal metallic grid that was then swiftly turned upside down. The latency to fall from the inverted grid was recorded for a maximum of 90 s. Each mouse was allowed to hold on to the inverted lid three times, and the longest latency to fall was recorded. Rotarod testing was performed for a maximum of 5 min, using 0.3 rpm/s, from 7 rpm to 28 rpm, constantly accelerating the rotarod apparatus (Ugo Basile 47,600 model). The time (in seconds) when the mouse fell from the rotating cylinder was recorded. Three trials were allowed, and the longest retention time was recorded for each mouse. The onset of neuromuscular impairment was defined as the age, in days, when the mouse exhibited the first failure in rotarod or grip strength performance.

### 4.4. Sample Collection for AXX71 Analysis

Animals at the end stage of the disease were deeply anaesthetized with Medetomidine 2 mg/kg, and Ketamin 150 mg/kg (~300 µL, IP), and decapitated. Brain and spinal cord tissues were rapidly removed and immediately frozen on dry ice and stored at −80 °C until they were homogenized in 4 volumes (weight/volume) of 0.1 N Hepes buffer (pH 7.0–7.5). At 0.5 and 4 h, after the last AXX71 IP administration, blood samples were collected in LiHe tubes to prevent coagulation (70 µL each), gently mixed, and immediately frozen in liquid nitrogen and stored at −80 °C or placed on wet ice and processed for analysis within 2 h. An amount of 70 µL from each sample was transferred into 1.5 mL tubes, containing 130 µL of 0.1 N Hepes buffer (pH 7.0–7.5), in double aliquot, for measuring the AXX71 antagonist concentration. The details are provided in Table 4.

### 4.5. AXX71 Measured in Blood, Brain, and Spinal Cord

Blood, brain, and spinal cord samples were analyzed using a method based on protein precipitation with acetonitrile, followed by LC/MS-MS analysis by an optimized analytical method. Since the stability of AXX71 in the blood, brain, and spinal cord is unknown, calibration standards (CS), and quality control samples (QC), were prepared and stored together with the study samples (it was assumed that this procedure would balance out any possible AXX71 degradation). Study samples, CS, QC, and blanks were spiked with an internal standard (IS—Rolipram) to improve the precision of the assay. Study samples were analyzed together with CS, QC, and blank samples (including also double blanks). From the calibration curve, the linear range of the analytical method was determined, and the lower and upper limits of the quantitation were specified. Details are provided in Table 5 and Table 6.

### 4.6. Spinal Cord Tissue Analysis

Spinal cords from SOD1-G93A mice, injected from postnatal day 98 with AXX71 (30 mg/kg), or A804598 (30 mg/kg) [31], were analyzed by Nissl staining for motor neuron count, by qRT-PCR for mRNA expression, and by Western blotting and confocal analysis for protein expression.

### 4.7. Nissl Staining

The number of motor neurons was determined on serial sections (30 μm thickness, one every eight sections) from the L3–L5 lumbar spinal cord segments from each mouse. The sections (*n* = 6/each mouse) were stained with 1% cresyl violet to detect the Nissl substance in the neuronal cells. The stained sections were gradually dehydrated in 50–100% alcohol, cleared in xylene, and coverslipped with Eukitt (Sigma-Adrich, Saint Louis, MO, USA). All sections were photographed at 20× magnification with a Zeiss Axioskop 2 microscope. Large neurons, with cell bodies ≥ 200 μm^2^ and well-defined cytoplasm, with nucleus and nucleolus, were then counted from the right and left ventral horns of each section using Neurolucida software (MBF Bioscience, Williston, VT, USA). The counts from 6 sections were averaged/each mouse.

### 4.8. Quantitative Real-Time PCR (qRT-PCR)

The lumbar spinal cords were lysed in TRIzol (Life Technologies, Monza, Italy), the RNA was extracted by sequential TRIzol/RNEasy Mini purification (Qiagen, Hilden, Germany), and reverse transcribed into cDNA with a SuperScript VILO cDNA synthesis kit (Life Technologies, Carlsbard, CA, USA). All qRT-PCR reactions were performed using 7900HT Fast Real-Time PCR (Applied Biosystems, Waltham, MA, USA), and carried out using SYBR Green (Life Technologies, Carlsbard, CA, USA) incorporated with gene-specific primers (listed below). The thermal conditions were the following: initial steps at 50 °C for 2 min, and 95 °C for 2 min, 40 cycles at 95 °C for 15 s, and 60 °C for 1 min, the melting curves were 95 °C 15 s, 60 °C 15 s, and 95 °C 15 s. Each reaction was performed in triplicate. Relative gene expression was calculated by ΔΔCT analysis relative to GAPDH expression levels. GAPDH: F, 5′ CATGGCCTTCCGTGTTTCCTA 3′, R, 5′ CCTGCTTCACCACCTTCTTGAT 3′; IL-1β F, 5′ GCAACTGTTCCTGAACTCAACT 3′, R, 5′ ATCTTTTGGGGTCCGTCAACT 3′; BDNF: F, 5′ CGGCGCCCATGAAAGAAGTA 3′, R, 5′ AGACCTCTCGAACCTGCCCT 3′; IL-10: F, GCATGGCCCAGAAATCAAGG 3′, R, GAGAAATCGATGACAGCGCC 3′; NOX2:F, 5′ TGAATGCCAGAGTCGGGATTT 3′, R, 5′ CCCCCTTCAGGGTTCTTGATTT 3′; Arg1: F, 5′-CCACGGTCTGTGGGGAAAGCCAAT-3′, R, 5′-CTGCCAGACTGTGGTCTCCACCCA-3′.

### 4.9. Western Blotting

Protein lysates were obtained by the homogenization of mice lumbar spinal cords in homogenization buffer (20 mM HEPES, pH 7.4, 100 mM NaCl, 1% Triton X-100, 10 mM EDTA) added with protease inhibitor cocktail (Sigma-Adrich, Saint Louis, MO, USA). After sonication, lysates were kept for 30 min on ice, and then centrifuged for 20 min at 14,000× *g* at 4 °C. Supernatants were collected and assayed for protein quantification with the Bradford detection kit (Bio-Rad Laboratories, Hercules, CA, USA). Separation of protein components was performed by Mini-PROTEAN^®^ TGX™ Gels (Bio-Rad, Hercules, CA, USA), followed by transfer onto nitrocellulose membranes (Amersham Biosciences, Buckinghamshire, UK). After saturation with 5% non-fat dry milk, blots were probed overnight at 4 °C with the specific primary antibodies: rabbit anti-P2X7 (1:500, Alomone Labs, Jerusalem, Israel); mouse anti-GFAP (1:500, Dako, Santa Clara, CA, USA); rat anti-CD68 (1:500, AbD Serotech, Oxford, UK); mouse anti-gp91phox (1:1000, BD Transduction, San Jose, CA, USA); rabbit anti-NFκB (1:500, Cell Signaling, Danvers, MA, USA); mouse anti-SOD1 (1:1000, Enzo Life Sciences, NY, USA); rabbit anti-LC3BI-II (1:500, Cell Signaling, Danvers, MA, USA); and mouse anti-SQSTM1/p62 (1:500, Abcam, Cambridge, UK). After incubation for 1h with specific HRP-conjugated secondary antibody, the blots were visualized using the ECL Advance Western blot detection kit (Amersham Biosciences, Buckinghamshire, UK). Signal intensity quantification was performed by Kodak Image Station analysis software. Values were normalized with mouse anti-GAPDH (1:2500, Calbiochem, San Diego, CA, USA).

### 4.10. Immunofluorescence and Confocal Microscopy

The mice were anaesthetized, as above, and transcardially perfused with 50 mL of PBS, followed by 4% paraformaldehyde at pH 7.4. Tissue samples were then post-fixed overnight in 4% paraformaldehyde in PBS, and then cryoprotected in 30% sucrose in PBS at 4 °C. Tissues were then stored at −80 °C. Spinal cords (L3-L5) were cut at a 30 μm thickness with a frozen microtome (see above). Double immunofluorescence analysis was performed according to the following procedure: a rectangle was drawn around the sections with a PAP pen (Sigma-Aldrich); after 1 to 2-h air drying, sections were blocked in PBS containing 10% normal donkey serum, and 0.3% Triton X-100, for 1 h at room temperature; spinal cord sections were incubated with either mouse glial fibrillary acidic protein GFAP (1:10, Dako), monoclonal rat anti-CD68 (1:100, AbD Serotech), mouse monoclonal anti-neuronal nuclei (NeuN) (1:200, Millipore, MA, USA) in PBS, supplemented with 0.3% Triton X-100 and 2% normal donkey serum, for 24 h at 4 °C. Sections were then washed with PBS and incubated with the appropriate fluorescent-conjugated secondary antibodies for 3 h at room temperature. The secondary antibodies used were: Cy2-conjugated donkey anti-mouse IgG (1:100, green immunofluorescence, Alexa, Molecular Probes Inc., Eugene, OR, USA), or Cy3-conjugated donkey anti-rat IgG (1:100, red immunofluorescence, Jackson Immunoresearch,). PBS washes (3 × 5 min) were performed, and sections were coverslipped with Fluoromount medium (Sigma-Adrich, Saint Louis, MO, USA). Immunofluorescence was analyzed by means of a confocal laser-scanning microscope (Zeiss, LSM700, Oberkochen, Germany) equipped with four laser lines: 405 nm, 488 nm, 561 nm, and 639 nm. The brightness and contrast of the digital images were equally adjusted using Zen software.

### 4.11. Statistical Analysis

The data on body weight, grip strength and rotarod performance were statistically analyzed with two-way ANOVA for repeated measures (time), and different groups (treatments), followed by a post hoc Sidack’s test, to compare the effects of the P2X7 antagonists, AXX71 or AXX13, with respect to the vehicle, at each time point. Statistical analysis was applied at the time points when all animals for each group were still alive, in order to maintain a balanced number of mice per group. The onset of neuromuscular impairment and the survival length were statistically evaluated by a log-rank test to compare probabilities. The Kaplan–Meier was used to analyze mice survival. The significance of differences among groups originating from the molecular experiments was analyzed by a one-way or two-way ANOVA, followed by a Sidack’s post hoc test. The Student’s *t*-test was used for independent comparison between two groups. Statistical analysis was carried out using the GraphPad Prism 8 software package, or SPSS 19.0 software (SPSS Inc., Chicago, IL, USA). A probability level of *p* < 0.05 was considered to be significant.

## Figures and Tables

**Table 1:** *In vitro* potency and selectivity profile.

	AXX71	AXX13
IC_50_ human P2X7 (nM)	14	6
IC_50_ mouse P2X7 (nM)	210	109
Selectivity		
(human P2X1,X2,X2/3,X3,X4,Y11; mouse P2X4)	inactive	inactive

Table 1 lists the *in vitro* potency and selectivity profiles of AXX71 and AXX13 compounds, as generated by Axxam SpA, Openzone.

**Table 2:** Pharmacokinetic analysis of AXX71 in mouse.

Time h	Animal ID	Blood ng/mL	Brain ng/g	Spinal Cord ng/g	Brain/ Blood Ratio	Spinal Cord/ Blood Ratio
0.5	D	956	747	496	0.78	0.52
0.5	E	1630	1650	931	1.01	0.57
0.5	F	1300	908	488	0.70	0.38
	**Mean**	**1295**	**1102**	**638**	**0.83**	**0.49**

Table 2 lists the concentration of AXX71 measured in the blood, brain, and spinal cord and expressed as ng/mL (in blood), or ng/g (in brain and spinal cord), after daily intraperitoneal dosing of the compound in C57BL/6J mice at 30 mg/kg for two weeks (vehicle: 30% Captisol*^®^* in PBS).

**Table 3:** Effects of P2X7 antagonists on ALS-related pathological features.

Parameter	AXX71 30 mg/Kg	AXX13 15 mg/Kg	A804598	BBG
**ARG1**	=	=	=	NA
**BDNF**	=	=	NA	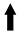
**CD68**	=	=	=	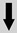
**GFAP**	=	=	=	=
**IL-1**	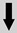	=	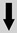	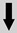
**IL-10**	=	NA	NA	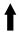
**LC3B-II**	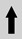	=	=	NA
**Motor behavior**	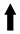	=	=	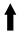
**Motor Neuron loss**	=	=	=	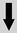
**NF-κB**	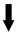	=	=	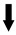
**NOX2**	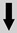	=	=	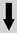
**P2X7**	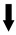	=	=	=
**SQSTM1/p62**	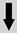	=	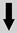	NA
**Survival**	=	=	=	=

Table 3 lists the effects induced in SOD1-G93A mice by the CNS’s most permeant P2X7 antagonists, AXX71, AXX13, and A804598, compared to the poorly CNS-permeant P2X7 antagonist, brilliant blue G (BBG). While BBG shows efficacy in several ALS-related parameters comprising motor impairment [28,29], A804598 affects only IL-1β and SQSTM1/p62 protein expression [31]. AXX71 administrated at 30 mg/kg/d shows higher effectiveness on the inflammatory (IL-1β; NOX2 and NF-κB) and autophagic parameters (SQSTM1/p62; LC3B-II), as well as on motor behavior with respect to AXX13 administrated at 15 mg/kg twice a day. The symbol ↑ refers to upregulation, ↓ to downregulation, and = indicates no change with respect to the vehicle-treated SOD1-G93A mice. NA means not applicable.

**Table 4:** Extraction Procedure.

Step	Process
1	Blood samples diluted 1:2.86 with 0.1N Hepes buffer (pH 7.0–7.5) (100 µL brain samples homogenized with 4 vol. 0.1 N Hepes buffer pH 7.4
2	200 μL Acetonitrile dispensed to double blanks
3	200 μL Internal Standard working solution in Acetonitrile (Rolipram; 100 ng/mL) dispensed to all other tubes
4	Tubes vortexed and mixed thoroughly
5	Tubes centrifuged for at least 10 min at approximately 3000× *g*
6	30 μL of the supernatant transferred to a tube containing 270 μL of 34% Acetonitrile
7	Tubes vortexed and mixed briefly
8	5 μL samples injected onto HPLC-MS/MS system for analysis

**Table 5:** HPLC Conditions.

Autosampler	CTC PAL	
Wash Solvent 1	Acetonitrile:Milli-Q Water (50:50)—0.1% Formic Acid	
Wash Solvent 2	Acetonitrile:Milli-Q Water (50:50)—0.1% NH3	
Typical Injection Volume	5 µL	
Chromatography System	API 4000	
Flow Rate	1.5 mL/min	
Analytical Column	Synergi RP-Max 30 × 2 mm, 4 µm	
Column Temperature	RT	
Run Time/Data Acquisition Time	2 min/2 min	
Mobile Phase A	0.1% Formic acid in water	
Mobile Phase B	0.1% Formic acid in acetonitrile	
Gradient	Time (min)	% Aqueous Phase
	0.00	95
	0.20	95
	1.50	5
	1.70	5
	1.80	95
	2.00	95

**Table 6:** MS/MS Conditions.

Mass Spectrometer				AB Sciex 4000			
Split Ratio				none			
Ionisation Interface and Temperature				TurboIonSpray™ at 650 °C			
Pause Time				5 msec			
Gas 1 Setting (Air)				60 psi			
Gas 2 Setting (Air)				40 psi			
Curtain Gas Setting (Nitrogen)				15			
Collision Gas Setting (Nitrogen)				6			
Analyte	Precursor ion (m/z)	Product Ion (m/z)	Dwell Time (msec)	Polarity	DP value	CE value	Typical R.T. (min)
AXX00179871	496.3	375.0	100	Positive	80	22	1.14
Rolipram	276.1	208.0	50	Positive	80	22	1.08

## Data Availability

Not applicable.

## Supplementary Materials

The following are available online at www.mdpi.com/article/10.3390/ijms221910649/s1.
