# Supplementary material for: Novel P2X7 Antagonist Ameliorates the Early Phase of ALS Disease and Decreases Inflammation and Autophagy in SOD1-G93A Mouse Model
Source: Int J Mol Sci. 2021 Sep 30;22(19):10649. doi: 10.3390/ijms221910649 (PMC8508678; doi:10.3390/ijms221910649)
Supplement: Supplementary file 1 [file ijms-22-10649-s001.zip › ijms-1382932-supplementary.pdf]

Table S1. Behavioural comparison between Captisol® solution-treated and untreated SOD1-G93A mice

	SOD1-G93A + Captisol® solution (vehicle of AXX71)	SOD1-G93A + Captisol® solution (vehicle of AXX13)	SOD1-G93A
**Onset (days)**	121.3 ± 7.5	123 ± 4.06	122 ± 5.6
**Survival (days)**	169.6 ± 9.76	171.4 ± 13.3	172 ± 7.3

Table S2. Biochemical comparison between Captisol® solution-treated and untreated SOD1-G93A mice

	SOD1-G93A + Captisol® solution	SOD1-G93A
**ARG1** *(RT-PCR)*	1.5 ± 0.8	1.4 ± 0.3
**BDNF** *(RT-PCR)*	0.8 ± 0.3	0.5 ±0.3
**CD68** *(WB)*	7 ± 0.8	5 ± 2
**GFAP** *(WB)*	5 ± 1.3	4.8 ± 0.8
**gp91^{phox}** *(WB)*	3 ± 0.2	4 ± 0.2
**IL-1β** *(RT-PCR)*	9 ± 0.3	9.5 ± 1.5
**IL-10** *(RT-PCR)*	0.6 ± 0.4	0.4 ± 0.2
**LC3B-II** *(WB)*	2.1 ± 0.2	3 ± 0.4
**Motor Neuron** *(IHC)*	0.34 ± 0.056	0.33 ± 0.12
**NOX2** *(RT-PCR)*	18 ± 0.05	16 ± 2
**NF-κB** *(WB)*	1.9 ± 0.25	2.2 ± 0.4
**P2X7** *(WB)*	3 ± 0.8	2.4 ± 0.1
**SQSTM1/p62** *(WB)*	1.7 ± 0.2	1.8 ± 0.5

The table lists the behavioural (Table S1) and biochemical (Table S2) parameters in Captisol® solution-treated *vs* untreated SOD1-G93A mice. In B values are represented as fold changes with respect to WT mice. Results for untreated SOD1-G93A mice derive from historical data collected in the laboratory. RT-PCR= real time-PCR; WB= western blot; IHC= immunohistochemistry.
